# Assessment of Human Respiration Patterns via Noncontact Sensing Using Doppler Multi-Radar System

**DOI:** 10.3390/s150306383

**Published:** 2015-03-16

**Authors:** Changzhan Gu, Changzhi Li

**Affiliations:** 1 Marvell Technology Group Ltd., 5488 Marvell Lane, Santa Clara, CA 95054, USA; E-Mail: cgu@marvell.com or changzhan.gu@ttu.edu; 2 Department of Electrical and Computer Engineering, Texas Tech University, Lubbock, TX 79409, USA

**Keywords:** radar, respiration, noncontact, vital sign

## Abstract

Human respiratory patterns at chest and abdomen are associated with both physical and emotional states. Accurate measurement of the respiratory patterns provides an approach to assess and analyze the physical and emotional states of the subject persons. Not many research efforts have been made to wirelessly assess different respiration patterns, largely due to the inaccuracy of the conventional continuous-wave radar sensor to track the original signal pattern of slow respiratory movements. This paper presents the accurate assessment of different respiratory patterns based on noncontact Doppler radar sensing. This paper evaluates the feasibility of accurately monitoring different human respiration patterns via noncontact radar sensing. A 2.4 GHz DC coupled multi-radar system was used for accurate measurement of the complete respiration patterns without any signal distortion. Experiments were carried out in the lab environment to measure the different respiration patterns when the subject person performed natural breathing, chest breathing and diaphragmatic breathing. The experimental results showed that accurate assessment of different respiration patterns is feasible using the proposed noncontact radar sensing technique.

## 1. Introduction

Respiration is the natural physiological activity that is regulated by the human brain to exchange oxygen and carbon dioxide with outside air. However, the respiration motion is not invariable, but changes in correspondence to different physical and emotional states, such as speaking, singing, fear, stress, *etc.* [1,2,3]. For example, “speech” is featured by shorter inspiration and longer expiration [2], and “fear” presents a shallow and rapid respiration pattern [3]. According to the National Center for Voice and Speech, improved breath control is not only essential to good singing, but also beneficial to the refinement of speech skills for those who frequently use their voice, e.g., lawyers and sales personnel. It is necessary to develop breathing skills to use the support from diaphragmatic or abdominal muscle for optimal singing/speech performance [4]. Chest breathing is human’s natural response to emergencies. When people feel stressed or anxious, they tend to increase the breathing rate and shift from diaphragmatic breathing to chest breathing, in order to take in extra oxygen to defend themselves [5]. However, chest breathing breaks the natural balance between oxygen and carbon dioxide in the body, which may lead to a variety of health problems such as musculoskeletal disorders [5]. In the modern, fast-paced society, more and more people are facing stress problem in both work and life. Diaphragmatic breathing is considered as a healthier way of breathing, which is marked by the abdominal expansion rather than the chest rib cage expansion [6]. It is a popular relaxation technique that helps stressed people to calm down.

To monitor the physical/emotional states of patients, assure the validity of breath control, and evaluate the efficacy of diaphragmatic breathing, it calls for accurate assessment of respiratory patterns at both chest and abdomen. The conventional respiration measurement techniques are mostly contact means, e.g., air mattress [7], the electrocardiogram (ECG) monitor [8], and the wearable systems [9], which bring discomfort to the patients and insufficient accuracy in respiration measurement. Moreover, the contact means may cause the subjects to breathe abnormally, while the spontaneous breathing is usually required for accurate assessment. Doppler radar technologies have been used for various applications, such as localization and mapping [10] and automotive obstacle detection [11]. Considering the disadvantages of the existing respiration assessment techniques, Doppler radar serves as a good alternative. Doppler radar provides a noncontact and more accurate approach to assess the respiration motion because it measures the whole chest wall or abdomen [12,13,14,15]. However, due to the high pass characteristic of the AC coupled baseband structure, the conventional alternating current (AC) coupled radar sensor is subject to signal distortion in measuring respiration, which has a short period of stationary moments after expiration in the respiration cycle [12]. To solve this problem, direct current (DC) coupled radar sensors have been proposed by researchers for accurate respiration measurement without signal distortions [16,17]. In the adaptive DC coupled radar sensor recently proposed by the authors [16,18], the baseband amplifier is not biased at a fixed point, but adaptively tuned through an external DC power source. In this way, the radar sensor is always able to measure respiration motion with sufficient high gain. Since the DC coupled baseband has “all-pass” architecture, it is possible for the radar sensor to accurately measure the complete respiration patterns without losing any signal information, which is a benefit to accurate assessment.

In this paper, a multi-radar system using two 2.4 GHz DC coupled radar sensors have been proposed for noncontact assessment of the respiration patterns at both chest and abdomen. The two radar sensors employ the same patch antennas but with different polarizations, in order to minimize the interference from each other. It is also feasible to use just one radar sensor with beam-scanning capability to simultaneously measure the breathing motions at multiple body locations [19]. The DC coupled radar system allows simultaneous assessment of the respiration motions at chest and abdomen with a very high accuracy.

Experiments were conducted in the lab environment to validate the proposed technique of using multi-radar system for noncontact assessment of different respiration patterns. Before the experiments, the subject person was trained to generate the diaphragmatic breathing and chest breathing. During the experiment, the subject laid on a bed in the supine position, with the two radar sensors facing his/her chest and abdomen, respectively. Different breathing patterns were collected and evaluated when the subject was asked to perform natural breathing, diaphragmatic breathing and chest breathing. The respiration assessment was also carried out for a group of people with different genders and physical characteristics. They were asked to perform natural breathing as well as breathing motions related to physical states of breath holding, cough and speaking. They were also asked to generate respiration patterns for the emotional states of anger and tenderness.

## 2. Radar Sensor System

Doppler radar provides a noncontact and noninvasive approach for physiological monitoring. It is especially suitable for some scenarios where contact means are prohibitive, such as burned patients. The radar noncontact approach potentially ensures more accurate assessment, because the target subject tends to have more spontaneous breathing patterns when they are not aware of the measurement device. Additionally, the contact measurement techniques may affect the spontaneous way of breathing by introducing discomfort. In radar respiration measurement, a single-tone carrier signal is transmitted to the subject’s chest or abdomen. The reflected signal from chest or abdomen is phase-modulated by the breathing motion. In this paper, the radar sensor was designed with quadrature architecture. So the measured signals at the baseband outputs are

(1) $$B{\left(t\right)}_{I}=A_{I}\cos\left[\text{θ}+4\text{π}x\left(t\right)/\text{λ}+\text{Δϕ}(t)\right]+DC_{I}$$

(2) $$B{\left(t\right)}_{Q}=A_{Q}\sin\left[\text{θ}+4\text{π}x\left(t\right)/\text{λ}+\text{Δϕ}(t)\right]+DC_{Q}$$

where θ is a constant phase offset determined by the initial position of the subject, *x*(*t*) is the breathing motion, Δϕ(*t*) is the residual noise including phase noise and other noise sources, *A_I_*/*A_Q_* are the amplitudes and *DC_I_*/*DC_Q_* are the DC offsets of the *I*/*Q* channels, respectively.

Several demodulation techniques, such as linear demodulation and arctangent demodulation, can be used to recover the breathing motion from the radar-received signal [20,21]. However, linear demodulation is not suitable for accurate respiration volume assessment because this technique is inherently based on small angle approximation, which does not provide efficient displacement accuracy [11]. Arctangent demodulation is accurate in displacement measurement and robust against the null point problem in radar respiration sensing [17,22]. However, it suffers from lack of accuracy if the radar sensor is AC coupled at baseband, since AC coupling makes it very difficult for accurate DC calibration due to the signal distortion.

**Figure 1:**
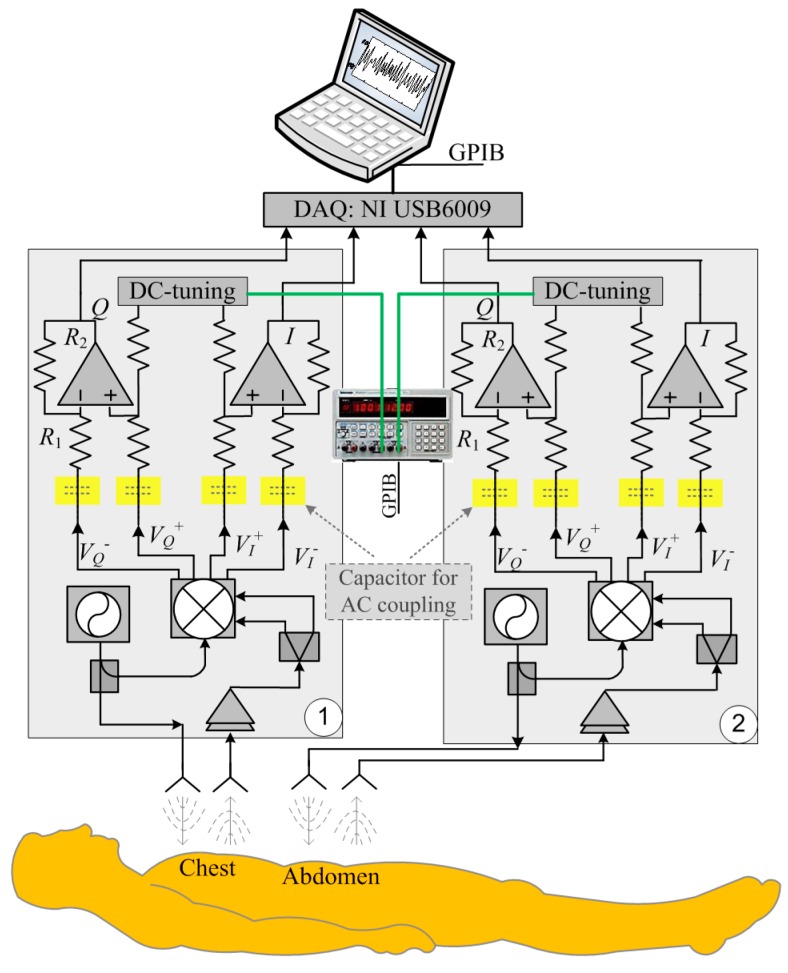
Block diagram of the DC coupled multi-radar system for respiration assessment at chest and abdomen.

Figure 1 illustrates the block diagram of the DC coupled multi-radar system that consists of two identical DC coupled radar sensors for measuring the respiration patterns at chest and abdomen. The DC coupled radars are configured with the fine-tuning adaptive feedback loop, which can dynamically adjust the DC offset to make sure the baseband circuitry can provide sufficient high gain as well as the optimal dynamic range [18]. The radar baseband *I*/*Q* outputs are captured by the data acquisition card (NI USB6009), which is connected to a laptop. The laptop also controls the power supply to perform adaptive DC tuning via a GPIB cable. The transmit power is 0 dBm, which is more than 20 dB lower than the Federal Communications Commission (FCC) regulations on transmit power in the 2.4 GHz ISM band [23]. The voltage-controlled oscillator (VCO) used in the system is from from Hittite Microwave and was designed for commercial applications and the spectrum fits within the ISM band from 2.40 GHz to 2.84 GHz. 

**Figure 2:**
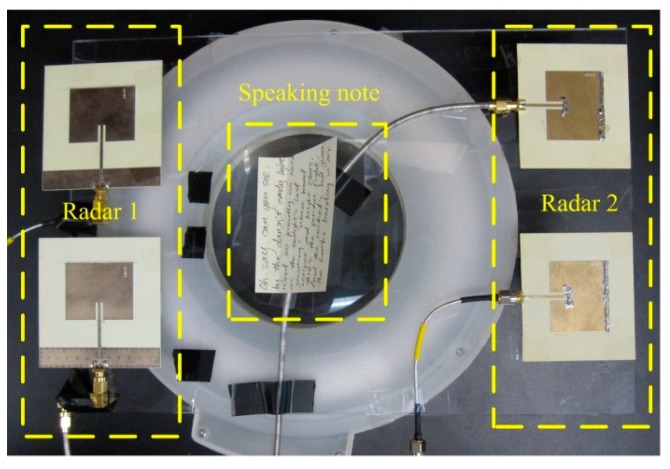
The antennas were placed in such a way that one radar used horizontally polarized antennas and the other radar used vertically polarized antennas. The speaking note was used to guide the subject during reading measurement.

Four patch antennas were used, since each radar sensor needs one antenna for transmitting and one antenna for receiving, respectively. Patch antenna was chosen for the following reasons: (1) it has a directional radiation pattern and reduce interferences from the back of the radar sensor; (2) it has a low cost, low profile, and is easy to fabricate; and (3) the planar patch antenna can potentially be integrated with the radar circuitry on the same printed circuit board. For each subject, the antennas’ positions were adjusted so that one radar radiates signals to the chest and the other radar radiates signals to the abdomen. The antennas were also placed in such a way that one radar sensor was horizontally polarized and the other one was vertically polarized, as shown in Figure 2. The two sensors have little interference to each other, due to the following reasons: (1) The two sensors use two free-running VCOs. Therefore, the carrier frequencies of the two sensors are slightly different. At 2.4 GHz carrier, the difference could easily be in the order of MHz. (2) The difference in carrier frequencies would be down-converted to the baseband. The sensor baseband was designed using operational amplifiers to have very narrow bandwidth of less than 1 MHz. Therefore, the interference could be easily rejected by the baseband circuitry. (3) Our radar operation is strongly based on coherent detection that effectively eliminates the impact of phase noise of each VCO on its corresponding receiver, while the two radar sensors are non-coherent and can hardly cause any significant impact on each other. (4) The different polarization of antennas further reduces the interference signal that leaks from one sensor to the other.

The above assumptions were experimentally verified by turning one of the sensors on and off. Little difference was observed on the signal detected by the other sensor. There might be occasional scenarios (approximately once in 5 min) where a strong spike/spur occurs at baseband output for a very short time interval. However, due to the free-running nature of the VCOs being used, the carrier frequencies tend to differ and therefore the spike/spur would disappear right away and can be eliminated in digital signal processing. The noise floor of the radar receiver plays an important role in determining the sensitivity. The factors contributing to the noise floor in CW radar sensor systems may include: (1) flicker noise—which dominates at very low frequencies; (2) white noise—which becomes the main noise source at frequencies higher than the flicker noise corner; (3) sampling rate *f*_s_ of the baseband ADC—the amplified white noise is folded into the frequency band from dc to *f*_s_ due to aliasing; and (4) the resolution bandwidth (*RBW*), which is determined by the time-domain window size of the Fourier transform—the narrower the *RBW*, the less noise energy is contained in a single point of the periodogram [24]. According to the analysis in [24], the input-referred noise floor can be quantified as:

Flicker noise = *KT***·***RBW***·***F*_flicker_(*f*) (3a)

White noise = *KT***·***RBW***·***F*_white_(*f*) **·** (*B*/*f*_s_) (3b)

where *F*_white_ is the noise figure at frequencies above the flicker noise corner frequency, *F*_flicker_ is the noise figure contributed by flicker noise, *B* is the equivalent noise bandwidth of the receiver, and *RBW* = 1/*TW* is the resolution bandwidth of the periodogram, which is determined by the Fourier transform window size *TW*. In the radar system being used in this paper, a typical window size of 10~20 s was used for real-time radar respiration sensing application. Assuming *F*_flicker_ = 40 dB at 1 Hz, *F*_white_ = 7 dB, *B* = 1 MHz, *RBW* = 0.1 Hz, and *f*_s_ = 20 Hz, the input-referred noise floor is:

Flicker noise = −174 dBm/Hz + 10 × log10(0.1 Hz) + 40 dB = −144 dBm
(4a)

White noise = −174 dBm/Hz + 10 × log10(0.1 Hz) + 10 × log10(1 MHz/20 Hz) + 7 dB = −130 dBm
(4b)

It should be noted that the relative strength of flicker noise and white noise is controlled by several parameters, such as *f*_s_ and *B*. If a high sampling rate, e.g., 10 kHz, was used, the input-referred white noise will be reduced to −157 dBm, making the flicker noise dominate the noise floor in the receiver chain.

## 3. Experiments

**Figure 3:**
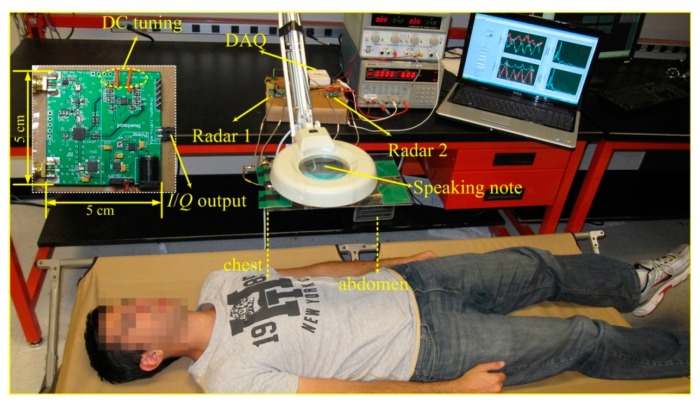
The Experimental setup of the DC coupled multi-radar system for assessment of different respiration patterns at chest and abdomen. Inset shows the photograph of the designed miniature DC coupled radar sensor.

Experimental evaluation was carried out in the lab environment for a group of ten healthy subjects including four females and six males. The anthropometric data of the study subjects are shown in Table 1. One of the subjects was trained to generate chest breathing and diaphragmatic breathing. And the remaining subjects were volunteers that were not trained in respiratory exercise. The experimental setup is shown in Figure 3. The subject lied in a folding bed in the supine position, with a distance of 50 cm from the patch antennas, as shown in Figure 3. The two radar sensors were placed on the table, connecting the antennas via coaxial RF cables. The data acquisition card (NI USB6009) collected the radar-measured signals, which were processed and displayed in real time by a LabVIEW program running on a laptop. Two power supplies were used to provide four channels of DC voltage to adaptively tune the DC offset for the two radar sensors. It should be noted that the DC tuning process should be carried out for every subject and every experiment. The DC offset was caused by the reflections from the stationary part of the subject body and the stationary objects around the subject. Each subject person has a different physical characteristics and each experiment has different surrounding environment, which leads to different DC offsets. In the newly developed radar sensor system, the function of automatic DC offset tuning has been integrated in the microcontroller in the baseband circuitry. That way, the system would automatically tune the DC offsets to adapt to different subjects.

Three experiments were conducted in this paper. They are described as follows.

**Table 1:** Anthropometric data of the study subjects.

Subject	Age	Sex	Chest Circumference	Abdominal Circumference
S1	26	Female	84 cm	72 cm
S2	30	Female	88 cm	73 cm
S3	25	Female	76 cm	64 cm
S4	23	Female	80 cm	67 cm
S5	27	Male	105 cm	91 cm
S6	26	Male	99 cm	89 cm
S7	22	Male	93 cm	85 cm
S8	27	Male	107 cm	93 cm
S9	31	Male	112 cm	90 cm
S10	22	Male	103 cm	90 cm

### 3.1. Breathing Type Discrimination

First, the different breathing types of natural breathing, chest breathing and diaphragmatic breathing were assessed. Only one subject participated in this experiment, as it would take a significant amount of time and effort for a normal subject to develop the desired breathing types by following the respiratory exercise [22,25]. It was difficult to train all the subjects that volunteered to participate in the experiment. To develop the diaphragmatic breathing, the subject was trained by putting one hand on the chest and one hand on the abdomen, and, with the help of hands to feel the chest and abdomen movement, the subject actively reduced the chest motion but breathed slowly through the abdomen [26]. After one week of exercise, the subject was able to breathe diaphragmatically without hands placed on the body. When people feel stressed or anxious, they tend to develop chest breathing by increasing the breathing rate and depth through the rib cage expansion [27]. The subject was asked to think of his stressed experience and mimicked the way he took breath during the stressed moment.

### 3.2. Physical Breathing Patterns

**Figure 4:**
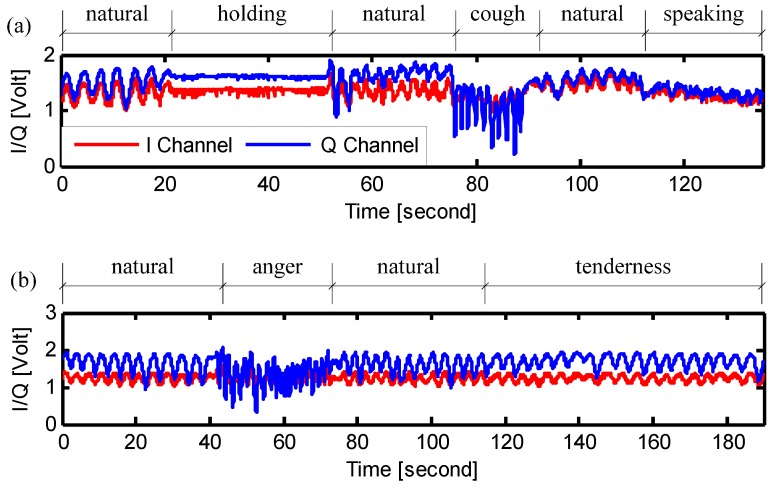
Signals measured at the abdomen of the subject showing the experimental procedures of (**a**) the assessment of physical breathing patterns; and (**b**) the assessment of emotional breathing patterns.

In the second experiment, the physical breathing patterns of the ten subjects were assessed. The procedure of the experiment is shown in Figure 4a. First, the subjects breathed normally at his/her natural rhythm. Then the subject was asked to hold the breathing as long as possible. After breath holding, the subjects started to breathe naturally again. The breath holding experiment is helpful in assessment of the radar performance in monitoring obstructive sleep apnea syndrome [27,28]. The subject was also asked to cough for a short period. This was to simulate the situations of how the breathing pattern may vary when the subject is sick. Finally, the subjects read the speaking note, which is the lyrics of the US national anthem, as shown in Figure 2 and Figure 3. This was to simulate the assessment of the respiration patterns in breath control exercise that is beneficial to improve speech and singing [2,4].

### 3.3. Emotional Breathing Patterns

In the third experiment, the emotional breathing patterns of the subjects were measured. It has been proven that the breathing patterns change in response to the emotional states, such as anger, anxiety, tenderness, sadness, *etc.* [3]. In other words, the breathing pattern is strictly related to the emotional state. It would be very interesting if we can tell the emotional state of a subject by remotely assessing his breathing patterns. This paper illustrates the preliminary results that show the feasibility of accurately measuring different respiration patterns via Doppler radar sensing, while more rigorous evaluation of the emotional states needs further research and experimental efforts by introducing spontaneously produced emotions.

The procedure of the experiment is shown in Figure 4b. The subjects started from the natural breathing and then were asked to reproduce two emotional sates of “anger” and “tenderness”. The state of “anger” was reproduced by giving the following breathing indications to the subjects: “breathe sharply through the nose; focus the eyes; tense the body as if you are ready to attack…” [1]. The subjects were then restored to natural breathing after the “anger” state. Finally, the subjects were asked to reproduce the emotional state of “tenderness” by following the indications of “keep body relaxed; put a little smile; breathe very evenly and gently” [1].

## 4. Results and Analysis

### 4.1. Breathing Type Discrimination

**Figure 5:**
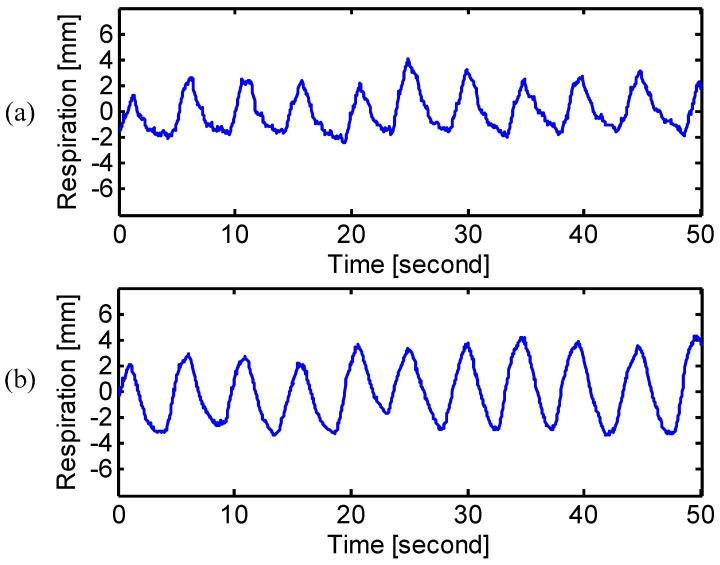
Radar measured signals at (**a**) chest and (**b**) abdomen when the subject was performing natural breathing.

**Figure 6:**
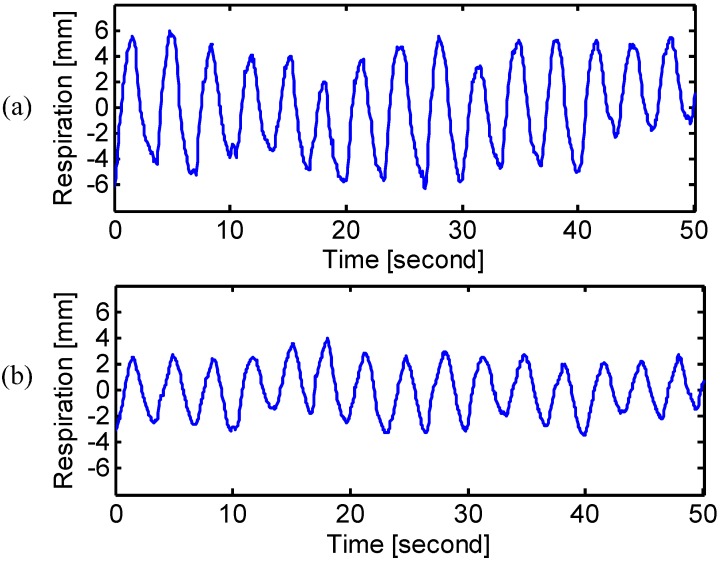
Radar measured signals at (**a**) chest and (**b**) abdomen when the subject was performing chest breathing.

**Figure 7:**
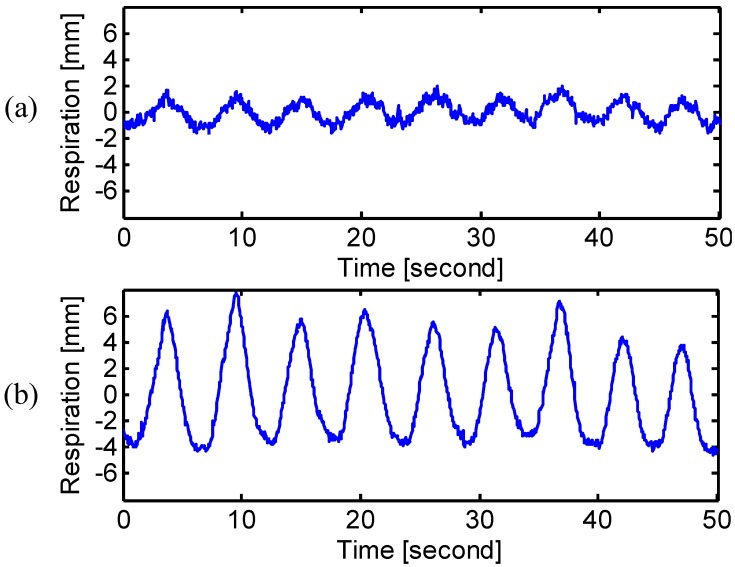
Radar measured signals at (**a**) chest and (**b**) abdomen when the subject was performing diaphragmatic breathing.

The experimental results of different breathing types are shown in Figure 5, Figure 6 and Figure 7. The measured frequency and the RMS amplitude of each breathing type were summarized in Table 2. For chest breathing, both the frequency and the chest amplitude significantly increased, which indicates the increase of tidal volume [25]. Figure 7 shows the radar measured respiration patterns for a subject who developed the way of breathing using the diaphragm. Compared with the natural breathing, most of the respiratory volume happened on the abdomen rather than the chest. The diaphragmatic breathing also finds therapeutic applications to treat diseases, such as asthma [22] and chronic obstructive pulmonary disease [25]. Radar respiration assessment is helpful to evaluate the respiratory exercise to develop the effective diaphragmatic breathing.

**Table 2:** Radar measured natural, chest, and diaphragmatic breathings.

Natural			Chest			Diaphragmatic		
Freq. [Hz]	Ch. (mm)	Ab. (mm)	Freq. (Hz)	Ch. (mm)	Ab. (mm)	Freq. (Hz)	Ch. (mm)	Ab. (mm)
0.22	1.46	2.17	0.30	3.35	1.81	0.18	0.60	3.37

### 4.2. Physical Breathing Patterns

**Figure 8:**
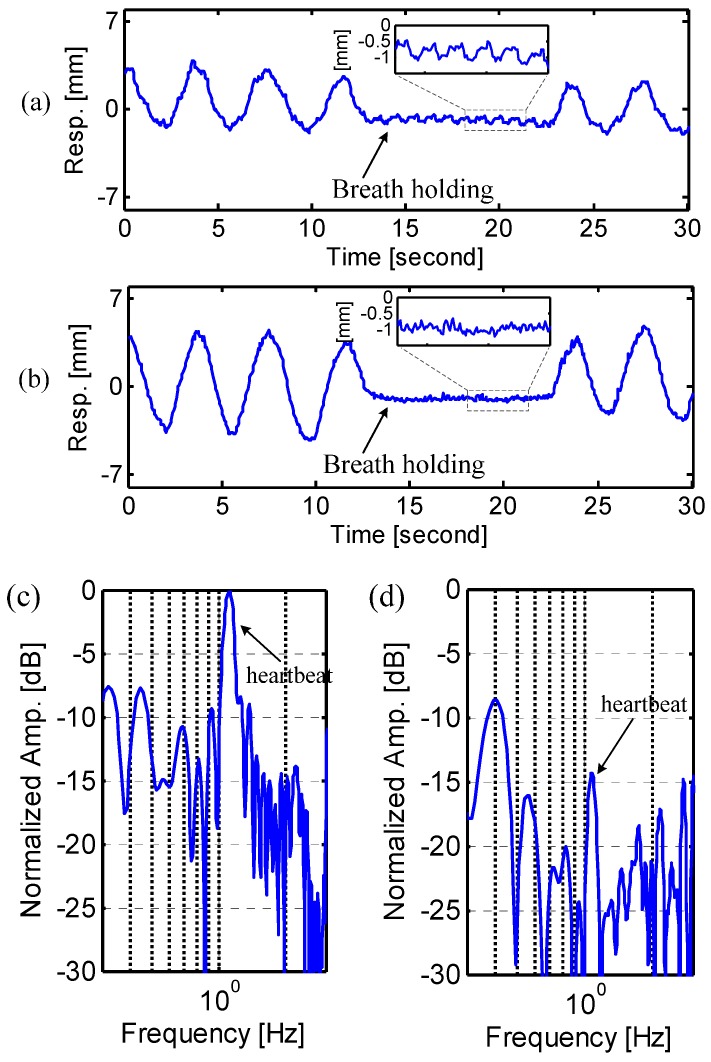
Radar measured respiration patterns from natural breathing to breath holding at (**a**) chest and (**b**) abdomen; (**c**,**d**) show the spectra of the breath holding at chest and abdomen, respectively.

Figure 8 shows the experimental results of breath holding. The respiration pattern at abdomen shows like a flat noise floor, but the pattern at chest shows modulated signals with lower amplitude compared to respiration. A further rigid analysis is presented in the frequency domain, as shown in Figure 8c,d. The modulated signals are actually the heartbeat that causes tiny vibrations on the chest wall and abdomen. It is seen that the heartbeat signal on the chest wall is 14.4 dB higher than on the abdomen. The heartbeat signal can serve to distinguish the breathing motions at chest and abdomen.

**Figure 9:**
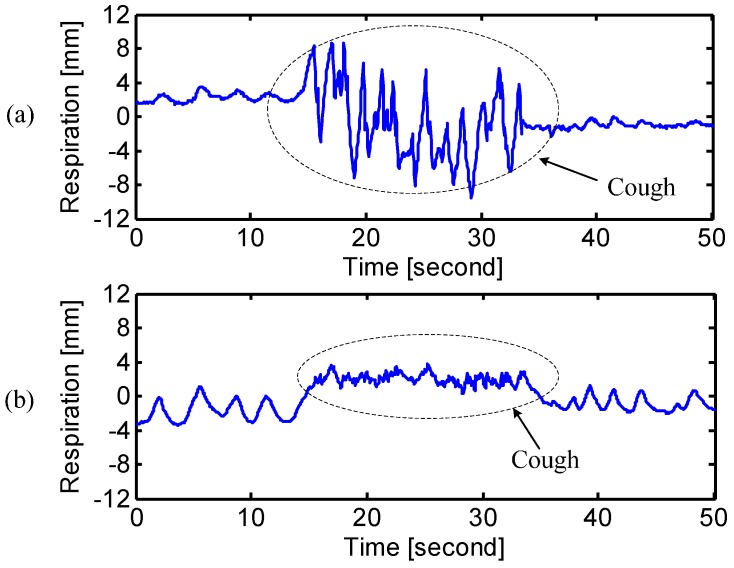
Radar measured respiration pattern of “cough” at (**a**) chest and (**b**) abdomen.

**Figure 10:**
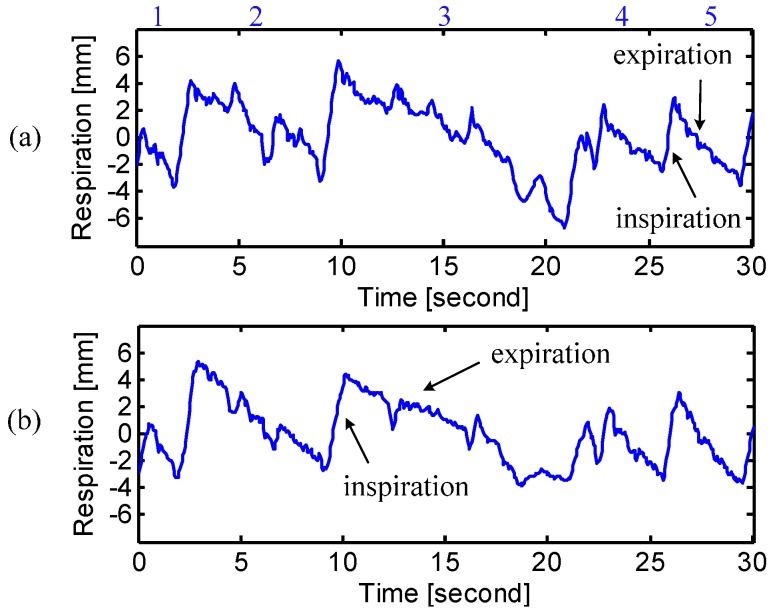
Radar measured respiration pattern of “speaking” overlaid with the words spoken at (**a**) chest and (**b**) abdomen. The pattern shows fast inspiration and slow expiration. The sections of words spoken are: “*1.* Oh; *2.* Say can you see by the dawn’s early light; *3.* What so proudly we hailed at the twilight’s last gleaming; Whose broad stripes and bright stars thru the perilous fight; *4.* O’er the ramparts we watched; were so gallantly streaming; *5.* And the rocket’s red...”

Figure 9 shows the situation when the subject coughed during breathing. It is illustrated that most of the cough strength happens on the chest rather than the abdomen. The radar sensing can be used for long-term home healthcare applications and tell symptoms ahead of time for better disease treatment. The experimental results when the subject was reading the lyrics of US national anthem were shown in Figure 10. The inspiratory and expiratory periods illustrated in Figure 10 represent the following lyrics overlaid with radar signals: “(1) Oh; (2) Say can you see by the dawn’s early light; (3) What so proudly we hailed at the twilight’s last gleaming; Whose broad stripes and bright stars thru the perilous fight; (4) O’er the ramparts we watched; were so gallantly streaming; (5) And the rocket’s red...”

Table 3 summarizes the measured data from all the ten subjects. It is observed that during the reading period, the subject raised his/her breathing strength at either chest or abdomen, compared to his/her corresponding natural breathing patterns. The increased breathing strength indicates the increased tidal volume during phonation [2]. It is seen in Figure 10 that the expiratory time increased during the speaking activity and the inspiration significantly decreased, which is the characteristic of the human phonation [2]. It is also seen from Table 3 that some subjects tend to depend on chest breathing to speak and some on abdomen breathing. The radar respiration assessment can help to study the interaction between speaking and breathing, so as to provide guidance in the breath control for improved singing and speech [4].

**Table 3:** Summary of Radar measured different respiratory patterns from the ten subjects.

Subject	Natural Breathing			Anger			Tenderness			Breath Holding (Second)	Speaking	
	Freq. (Hz)	Ch. RMS (mm)	Ab. RMS (mm)	Freq. (Hz)	Ch. RMS (mm)	Ab. RMS (mm)	Freq. (Hz)	Ch. RMS (mm)	Ab. RMS (mm)		Ch. RMS (mm)	Ab. RMS (mm)
S1	0.22	1.25	2.17	---	2.09	1.44	0.17	1.58	1.28	45.7	0.95	2.69
S2	0.19	0.76	2.95	---	4.45	3.08	0.20	1.12	2.98	47.3	2.05	3.99
S3	0.21	1.04	2.82	---	1.18	1.14	0.16	1.54	2.72	27.6	1.47	4.12
S4	0.19	0.80	2.45	---	1.33	2.90	0.14	0.91	2.25	24.5	2.84	2.36
S5	0.30	1.03	1.98	---	0.97	1.52	0.15	0.64	2.23	31.1	1.17	2.31
S6	0.29	0.65	1.71	---	5.30	2.30	0.22	0.57	1.13	30.5	1.54	3.72
S7	0.22	0.35	2.93	---	3.61	4.23	0.18	0.46	1.81	27.9	4.01	2.95
S8	0.19	1.38	3.23	---	1.02	3.97	0.15	0.55	2.94	38.5	1.31	4.01
S9	0.24	0.34	2.52	---	0.96	1.87	0.11	0.99	3.40	25.3	1.83	2.79
S10	0.26	1.20	1.75	---	2.09	1.82	0.18	1.09	1.87	33.0	1.64	2.31

### 4.3. Emotional Breathing Patterns

**Figure 11:**
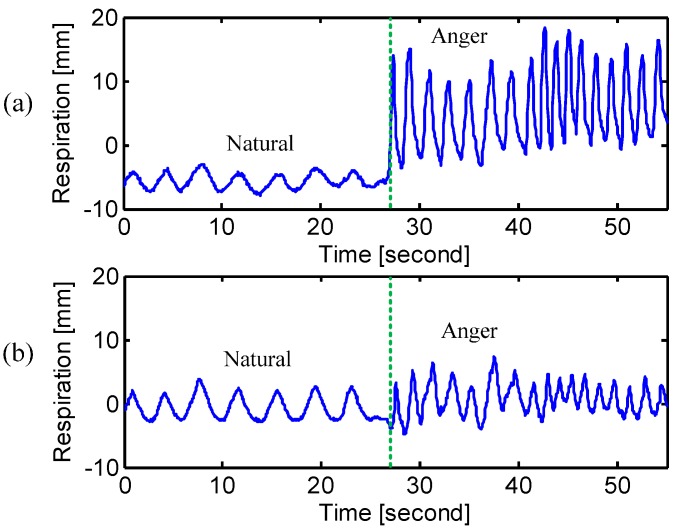
Radar measured respiration patterns of “anger” at (**a**) chest and (**b**) abdomen. The dashed line indicates the transition from natural breathing to the emotional state of “anger”.

**Figure 12:**
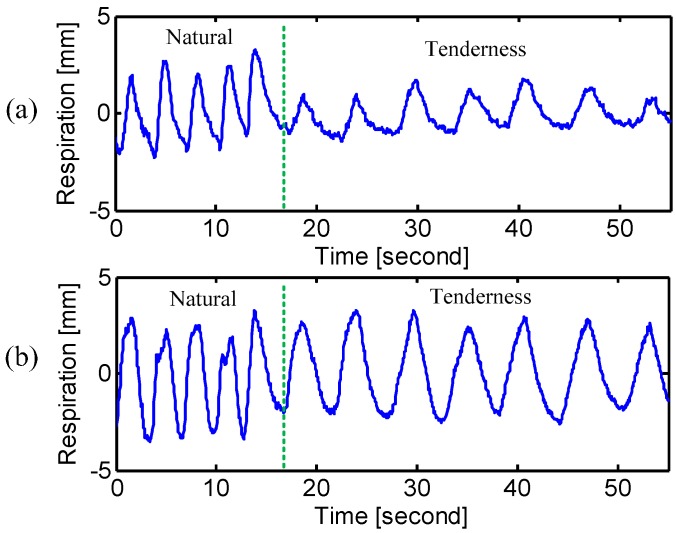
Radar measured respiration patterns of “tenderness” at (**a**) chest and (**b**) abdomen. The dashed line indicates the transition from natural breathing to the emotional state of “tenderness”.

The experimental results for emotional breathing patterns are summarized in Table 3. Since the “anger” state is not regular in rhythm, the factor of frequency is not included. Figure 11 and Figure 12 show one case of the emotional breathing patterns. It is seen from Figure 11 that the subject tends to expand his chest rib cage more than the abdomen during the state of “anger”. However, it should be noted that different people have different breathing patterns for the “anger” state, which can be seen from Table 3. Moreover, it is found that the subject exhibited different levels of respiratory volume depending on the different degrees of anger. Figure 12 illustrates the emotional state of “tenderness”, where the respiration shows a slow and even pattern [1,3]. Since the emotional states are strictly related to the respiration pattern, the radar respiration assessment has the potential ability to identify the unknowing subject’s emotional state.

## 5. Conclusions

A noncontact technique for accurate assessment of human respiratory patterns was presented in this paper using a DC coupled multi-radar system. The proposed radar is able to retain the entire signal integrity for slow respiration motion. Three different breathing types, *i.e.*, natural breathing, chest breathing and diaphragmatic breathing, were assessed for a trained subject. Ten subjects were assessed for their different physical and emotional breathing patterns. It is found that the radar technique is able to accurately identify all the different respiration patterns. It serves well as an assessment technique in respiratory exercise to develop diaphragmatic breathing and breath control practice for good singing and speech.
